# Royal South Hants Infirmary, Southampton

**Published:** 1895-09-28

**Authors:** 


### The Institutional Workshop.

### WITHIN THE HOSPITALS.

### ROYAL SOUTH HANTS INFIRMARY, SOUTHAMPTON.

(By Our Special Commissioner.)

It will be in the remembrance of some of our readers that we published a report on this institution, the result of an inspection in 1892, *vide* The Hospital, July 23rd, 1892, page 285. At that time we pointed out that a good part of the present buildings are old and out of date, although efforts have been made to improve the sanitary condition of this portion of the institution and in various ways to bring it more up to modern requirements. It was desirable for the credit of Southampton that much of it should be pulled down and rebuilt. Our report showed that several of the wards which contained beds were ill-adapted to the successful treatment of severe cases. Unfortunately, despite the marked progress which Southampton has made in recent years, the infirmary committee and governors have not yet reconstructed the buildings of this institution, as they undoubtedly ought to do. Having regard to the development of the traffic in the docks, and the consequent increase both in the working population and in the number of accident cases, a new wing is urgently needed, but has not yet been erected, in connection with the Royal South Hants Infirmary. The delay in commencing this work is the more remarkable from the fact that the hospital has a large amount of invested funds, that it is well supported, and that there is evidence that the committee, or many of them, take a warm interest in the institution. In these circumstances, seeing the urgent need which undoubtedly exists for further and adequate accommodation in connection with the infirmary, we hope that the plans of the architect, after being carefully revised by the medical staff, with the advice of a skilled expert, may be accepted, and that the building operations will be commenced forthwith. Not only the people of Southampton, but those resident in the surrounding districts seem to take an interest in the well-being of the Royal South Hants Infirmary, and we are confident that an abundance of funds might be secured if the proper steps were taken to bring the facts to the notice of those who are able to contribute. As we said on a former occasion, it is essential to the credit of Southampton and its growing importance as a seaport that the Royal South Hants Infirmary should be promptly made to comply with the advanced rules of hospital construction. Any further delay must seriously reflect not only on the capacity of the committee, but upon the intelligence and public spirit of the people of Southampton.

#### The Advantages of a Good Matron.

Since 1892 there has been a change in the office of matron, and Miss Mollett, who at present holds that office, is one of the most experienced and able of modern lady superintendents. The appearance and condition of the Southampton Infirmary at the present time testifies to the influence for good which a really able lady superintendent can exercise in the administration of a hospital where faulty construction and other causes tend to diminish efficiency. We were struck with the marked improvements which Miss Mollett has introduced. She has convinced the committee of the force and truth of what we wrote in 1892 as to the impropriety of treating patients in certain of the wards then occupied, and some of them are now closed altogether to patients. The children's ward has been moved to another part of the building, and its condition and the aspect of the children testifies to the skill of the present administration and to the advantages derived from the improved hygienic conditions. It requires a good deal of courage to advocate the closing of wards in any hospital, where the medical staff are wisely jealous of any reduction in the number of beds which may be available for the treatment of cases. This difficulty Miss Mollett has overcome with courage and success, and in overcoming it she has done much to improve the whole atmosphere of the institution and to materially hasten the time when adequate buildings for the reception of patients will be erected at Southampton. But her presence has done more than this, for Miss Mollett has succeeded in impressing upon the place a general air of robust healthiness which must have tended to increase the happiness and comfort not only of the patients but of the staff, and so to make for efficiency. The atmosphere of the wards was much better than at the time of our last visit, and the faces of the patients and the appearance of the nurses proved how welcome the changes referred to have in practice been. It has been our custom to inspect a number of institutions and hospitals every year during upwards of a quarter of a century, and we are now able to realise from the general appearance and aspect of a given institution the relative position which it occupies in regard to general management and administration. No one of experience can visit this institution to-day without realising the enormous advantages of a good matron to everybody connected with a hospital, and Miss Mollett deserves the grateful thanks, as she has no doubt already won the warm appreciation, of the people of Southampton.

#### A New Wing Needed.

As we have already said, no time should be lost in taking steps to erect a new wing. The site is ample, and presents no difficulties whatever; on the contrary, it lends itself to the needed extension. We understand that plans have been prepared, but before they are finally accepted we would strongly urge those responsible to submit them to some recognised authority who has a knowledge of all the recent developments in modern hospital construction, so that the new wing may be a model of perfection, and reflect honour not only upon the institution, but upon the seaport where it is situated. Unlike many institutions where capital is scanty, the Royal South Hants Infirmary is in the happy position of possessing invested funds, and which have been materially added to in recent years, and amount altogether to some £50,000. The ordinary expenditure amounts to considerably less than £6,000 per annum, so that an invested endowment amounting to £20,000 would be adequate to provide for any temporary diminution in income due to exceptional causes. It follows that a sum of £30,000 might be placed to the credit of the building fund without hesitation. The balance required to erect the new wing would be, we are confident, easily raised with the assistance of the representatives of the large railway and shipping companies who are so closely identified with the work of the hospital, and the growing prosperity of Southampton. The laundry buildings are hopeless from an administrative, and from every point of view. It is quite impossible for the matron to hope to get her linen into anything like proper order until she is provided with an adequate laundry properly constructed and fitted. In this connection we would strongly advise the committee to send the matron and architect to see how the laundries are conducted at the leading Scotch hospitals in Glasgow and Edinburgh. There can be no doubt that the Scotch system of placing at the head of the laundry a woman of education and special intelligence is attended, with the happiest results. There is certainly as much dirt and drawback, so far as laundry operations are concerned, in Glasgow and at the Edinburgh Royal Infirmary, as in London, yet the colour and appearance of the linen of the Scotch hospitals is splendid compared with that which they present in the metropolitan hospitals, or even in a county infirmary like that at Southampton, where, as we have said before, the laundry arrangements are hopeless at thep resent time.

### Final Points.

In the course of our inspection we could not fail to be struck with the wonderful cleanliness of everything everywhere. The condition of the baths and the lavatories and floors was highly commendable. Miss Mollett has also turned her attention to the bedding and bedsteads, which were obsolete when she took office. Thanks to her energy new bedsteads have now been introduced, and the patients have to thank her for much additional comfort, and the medical staff for improvements in the hygienic conditions, which must tend to expedite the recovery of the cases under their care. But to our mind the greatest triumph achieved by Miss Mollett is the closing of certain wards which were formerly occupied by patients, although such a course was hardly justifiable. Where it has not been found possible to entirely close an old ward ill-adapted to the treatment of disease, the number of beds have been reduced. The atmosphere and hygiene has in consequence been immeasurably improved. Altogether, we feel justified in stating that those hospital committees or governors who may be dissatisfied with the condition of the hospital buildings with which they may be connected should certainly pay a visit to the Royal South Hants Infirmary, so as to see what it is possible for an able administrator to do with the least promising materials. All honour to Miss Mollett, and all honour to her committee and the medical staff of the Royal South Hants Infirmary, who have had the wisdom to recognise the soundness of her representations, and to give her a free hand to raise this institution to its present state of excellence throughout—so far as excellence is possible in the absence of adequate ward accommodation, a new laundry, and a proper nurses' home. The last is urgently called for, and we should like to see some wealthy resident of Southamptom come forward and give the necessary money to defray the cost of such a building.

